# Effects of Cardiolipin on the Conformational Dynamics of Membrane-Anchored Bcl-xL

**DOI:** 10.3390/ijms22179388

**Published:** 2021-08-30

**Authors:** Vivek Tyagi, Victor Vasquez-Montes, J. Alfredo Freites, Alexander Kyrychenko, Douglas J. Tobias, Alexey S. Ladokhin

**Affiliations:** 1 Department of Materials Science and Engineering, University of California, Irvine, CA 92697, USA; vivekt@uci.edu; 2 Department of Biochemistry and Molecular Biology, The University of Kansas Medical Center, Kansas City, KS 66160, USA; vvasquez@kumc.edu (V.V.-M.); alexander.v.kyrychenko@univer.kharkov.ua (A.K.); 3 Department of Chemistry, University of California, Irvine, CA 92697, USA; jfreites@uci.edu; 4 Institute of Chemistry & School of Chemistry, V. N. Karazin Kharkiv National University, 4 Svobody Square, 61022 Kharkiv, Ukraine

**Keywords:** Bcl-xL, BCL-2 proteins, BH3-binding site, cardiolipin, apoptotic regulation, protein–membrane interactions, molecular dynamics simulations

## Abstract

The anti-apoptotic protein Bcl-xL regulates apoptosis by preventing the permeation of the mitochondrial outer membrane by pro-apoptotic pore-forming proteins, which release apoptotic factors into the cytosol that ultimately lead to cell death. Two different membrane-integrated Bcl-xL constructs have been identified: a membrane-anchored and a membrane-inserted conformation. Here, we use molecular dynamics simulations to study the effect of the mitochondrial specific lipid cardiolipin and the protein protonation state on the conformational dynamics of membrane-anchored Bcl-xL. The analysis reveals that the protonation state of the protein and cardiolipin content of the membrane modulate the orientation of the soluble head region (helices α1 through α7) and hence the exposure of its BH3-binding groove, which is required for its interaction with pro-apoptotic proteins.

## 1. Introduction

Apoptotic dysregulation is a common feature of many diseases and contributes to neurodegeneration, immunodeficiency, and cancer [1,2,3]. The anti-apoptotic protein Bcl-xL is a crucial component of this pathway as it blocks mitochondrial outer membrane permeabilization (MOMP), thereby preventing cell death [4,5]. This is achieved by binding the pore-forming proteins BAX and BAK, resulting in non-productive oligomers that can no longer participate in promoting apoptosis [6,7].

**Figure 1:**
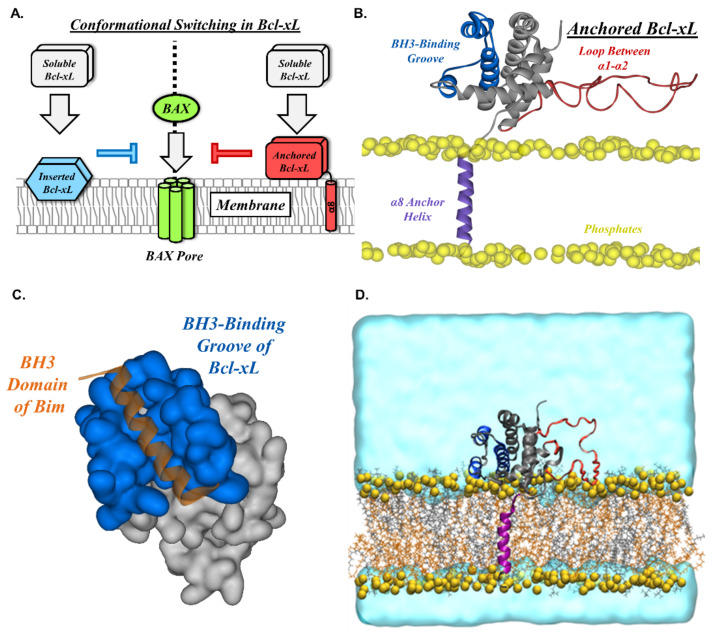
Membrane associated Bcl-xL. (**A**) According to the “embedded together” model, the interactions between the anti-apoptotic protein Bcl-xL and its pro-apoptotic targets (e.g., pore-former BAX) require their transition to the mitochondrial outer membrane [9]. Two different membrane-integrated Bcl-xL conformations have been identified: membrane-inserted and membrane-anchored Bcl-xL. The former is characterized by extensive refolding [15,16], while the latter retains the fold of the soluble state (aside from the released α8 anchor helix) [12,13,14]. Here, we focus on the membrane-anchored conformation of Bcl-xL and the influence of cardiolipin and protonation on its conformational dynamics. (**B**) The initial configuration of membrane-anchored Bcl-xL used in the all-atom molecular dynamics simulations. The protein is shown in secondary structure representation. The soluble head region (helices α1 through α7) is shown in grey with its BH3-binding groove highlighted in blue. The loop connecting α1 and α2 is shown in red and the α8 anchoring helix is shown in purple in a transmembrane configuration. The lipid phosphate phosphorus atoms are shown as yellow spheres. The rest of the simulation system has been removed for clarity. (**C**) A surface representation of the BH3-binding groove of Bcl-xL (blue) bound to the BH3 domain of Bim (orange), PDB ID: 3FDL. (**D**) A cut-away representation of the complete simulation system. The protein and lipid phosphate phosphorus atoms are rendered as in (**B**), the rest of the lipid molecules are shown in licorice representation (POPC, grey; TOCL, orange), and water molecules are rendered as slabs in cyan.

According to the “Embedded Together” model of MOMP regulation, the interactions between Bcl-xL and its target proteins occur on the mitochondrial outer membrane as this is where pore-forming proteins attach to initiate an apoptotic process (Figure 1A) [8,9]. This requires the transition of Bcl-xL from an inactive state in the cytosol to an active state in the bilayer [10,11]. Two different membrane-integrated forms of Bcl-xL have been identified in vitro: (1) an anchored conformation in which the C-terminal helix of Bcl-xL serves as a transmembrane anchor, while the remaining helices retain their soluble fold (Figure 1A, red) [12,13,14], and (2) a membrane inserted conformation that involves the interaction of several Bcl-xL regions with the bilayer and extensive refolding, which leads to the release of the N-terminal BH4 helix (Figure 1A, blue) [15,16].

The specific roles of the different membrane-integrated conformations of Bcl-xL and their anti-apoptotic functions remain unclear; nevertheless, each has been linked to a different mode of apoptotic regulation. In the case of the anchored conformation, the lack of the refolding of the soluble head region (helices α1 through α7), as compared to the fold in the solution, results in an intact BH3-binding groove [12,13,14] (Figure 1B,C). BH3 domains are conserved regions in Bcl-xL homologs, such as its targets BAX and BAK, that are used to recognize and bind other apoptotic regulators [8,10,11]. Meanwhile, the global refolding in the inserted conformation is likely to disrupt the BH3-binding groove in Bcl-xL, suggesting an alternative mechanism to bind and inhibit its target proteins.

The lipid composition of mitochondrial membranes has a prominent effect on the membrane interactions and refolding of Bcl-xL. The mitochondria-specific lipid cardiolipin is of particular importance because its abundance in mitochondrial membranes presents a preferential distribution, with significant enrichment at mitochondrial contact sites [17,18,19]. These cardiolipin-rich regions are often referred to as hotspots for apoptotic regulation and under non-apoptotic conditions they contain an approximate 30% cardiolipin fraction compared to the average 4% of the mitochondrial outer membrane [20,21]. Additionally, apoptosis-related increases in cardiolipin concentration at the mitochondrial outer membrane (redistributed from the inner mitochondrial membrane) [20,21] could further modulate Bcl-xL interactions and conformations.

To better understand how the enrichment of cardiolipin affects the membrane-anchored form of Bcl-xL (Figure 1B), we performed all-atom molecular dynamics (MD) simulations (Figure 1D) in the presence or absence of cardiolipin in a phosphatidylcholine matrix (2:1 PC:CL or 100% PC, respectively). Simulations were performed with all acidic and histidine sidechains either protonated or deprotonated due to the significance of the protein protonation state on the Bcl-xL membrane interactions [15,16]. The results from these simulations indicate that cardiolipin and protonation modulate the dynamics of the α1-α2 loop as well as the orientation of the soluble head region of membrane-anchored Bcl-xL.

## 2. Results

### 2.1. Orientation of the Soluble Head and Accessibility of the BH3-Binding Groove

**Figure 2:**
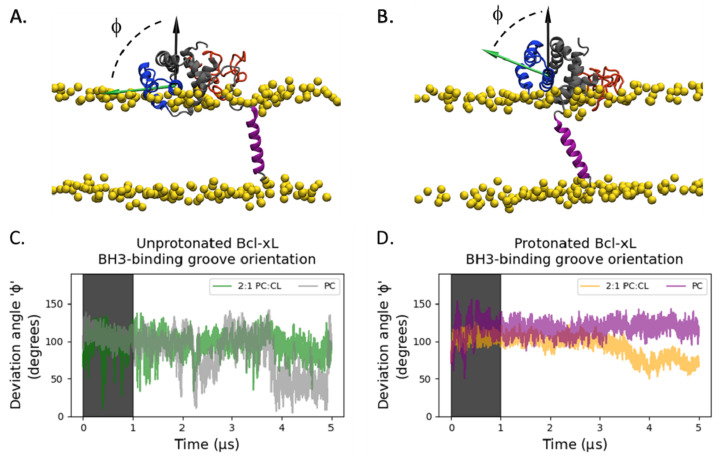
Accessibility of the Bcl-xL BH3-binding groove. (**A**) Configuration snapshot corresponding to a BH3-binding groove vector (green arrow) with a deviation angle from the membrane normal (black arrow), ϕ, of greater than 90°, suggesting occlusion of the BH3-binding groove (color scheme as in Figure 1B). (**B**) Configuration snapshot corresponding to a groove vector with a shallower than 90° deviation angle from the membrane normal, suggesting an accessible BH3-binding groove (color scheme as in Figure 1B). BH3-binding groove deviation angle as a function of time for (**C**) unprotonated Bcl-xL in a 2:1 POPC:TOCL lipid bilayer (green) and POPC lipid bilayer (grey), and (**D**) protonated Bcl-xL in a 2:1 POPC:TOCL lipd bilayer (yellow) and POPC lipid bilayer (purple). The shaded regions (time < 1 μs) represent the non-stationary portion of the trajectories and were not included in further analyses (see also Supplemental Figure S1).

To evaluate the effect of the mitochondrial specific anionic lipid cardiolipin (1,1,2,2,-tetraoleoyl-cardiolipin, TOCL) and protein protonation state on the conformational dynamics of membrane-anchored Bcl-xL, we performed four all-atom MD simulations (see Table 1 and Figure 1D): protonated and unprotonated Bcl-xL in a POPC (palmitoyl-oleoyl-phosphatidylcholine) lipid bilayer, as well as protonated and unprotonated Bcl-xL in a 2:1 POPC:TOCL lipid bilayer. Analysis of the Bcl-xL soluble head orientation and corresponding accessibility of its BH3-binding groove were carried out by defining a binding groove vector (see Materials and Methods) and monitoring its orientation with respect to the membrane normal, as illustrated in Figure 2.

The deviation of the BH3-binding groove vector from the membrane normal, depicted in Figure 2A,B, is shown as a function of time in Figure 2C,D. Deviation angles above 90° (Figure 2A) are indicative of a partially occluded binding site and values below 90° (Figure 2B) are indicative of a more accessible BH3-binding groove. The unprotonated 2:1 POPC:TOCL simulation shows large fluctuations with the deviation angles primarily occupying angles between 90° and 110° (Figure 2C). The unprotonated POPC simulation has a similar behavior until around 3.75 μs, where it shows a sharp transition from values above 90° to values centered approximately at 40°, this configuration is sampled for about 1 μs before transitioning back to a value above 90° (Figure 2C). Fluctuations are clearly smaller in the protonated simulations (Figure 2D) with both simulations exhibiting similar behavior until 2.75 μs, when the cardiolipin-containing simulation gradually converges to lower values under 90° through a sustained rotation of the soluble head over a period of 1 μs (Figure 2D).

**Figure 3:**
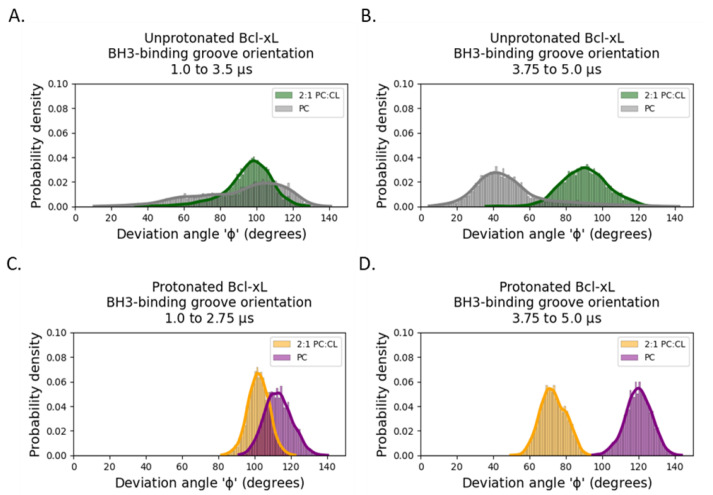
Orientational configurations of the BH3-binding groove. The changes in the distributions of the BH3-binding groove deviation angle from the membrane normal over two separate time intervals indicate that the presence of cardiolipin narrows the configurational space sampled by the soluble head and that both protonation of Bcl-xL and cardiolipin favors orientations of the soluble head, consistent with accessible configurations of the BH3-binding groove. (**A**,**B**) Binding-groove deviation angle distributions for unprotonated simulations based on stationary portions of unprotonated PC system. (**C**,**D**) Binding groove deviation angle distributions for protonated simulations based on stationary portions of protonated 2:1 PC:CL system. All angle histograms are colored as in Figure 2C,D.

Regarding the binding groove deviation angle in Figure 2, we considered the corresponding deviation angle distributions over two distinct time intervals exhibiting steady-state behavior (see Figure 3). The distributions for both unprotonated systems remain broad and overlapping over both sampled time intervals (Figure 3A,B). Comparison between the sampled time intervals suggests that in the unprotonated POPC simulation, the BH3-binding groove deviation angle is sampling the entire allowed configurational space over the microsecond timescale, while in unprotonated POPC:TOCL simulation, the sampling of large deviation angles is favored. Only the protonated POPC:TOCL simulation shows a separate narrow distribution of deviation angles under 90° (Figure 3D).

Taken together, these results suggest that protonation of Bcl-xL titratable residues (ten aspartic acids, twenty-one glutamic acids, and four histidines) modulates the conformational dynamics of the Bcl-xL soluble head region in the membrane-anchored configuration. Furthermore, the presence of cardiolipin promotes the re-orientation of the soluble head in protonated Bcl-xL towards a configuration that exposes the BH3-binding groove.

### 2.2. Conformational Dynamics of the α1-α2 Loop

**Figure 4:**
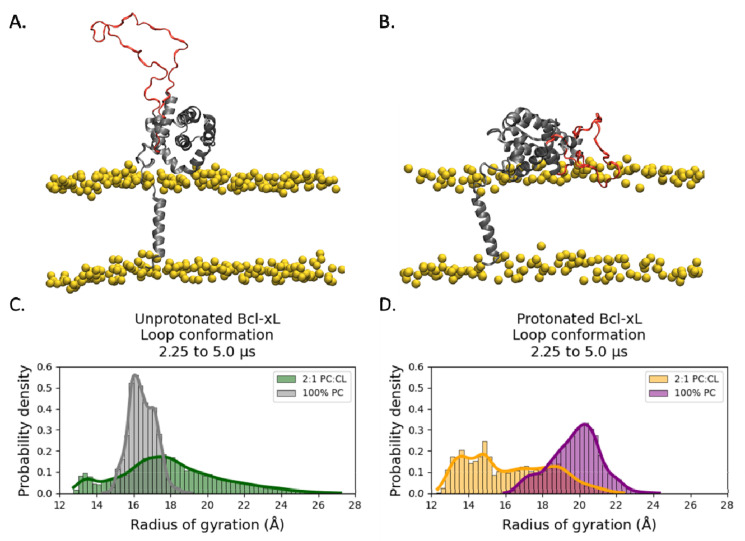
Conformation of the α1-α2 loop. (**A**) Configuration snapshot corresponding to an extended loop conformation in the unprotonated POPC:TOCL system. (**B**) Configuration snapshot corresponding to a compact loop conformation in the protonated POPC:TOCL system. The protein is shown in secondary structure representation colored gray, except for the α1-α2 loop which is shown in red. The lipid phosphate phosphorus atoms are shown as yellow spheres. The rest of the simulation system has been removed for clarity. The α1-α2 loop radius of gyration is broadly distributed in the POPC:TOCL systems in comparison to POPC, suggesting that the presence of cardiolipin confers more flexibility to the loop. The comparison between the radius of gyration distributions in the unprotonated (**C**) and protonated (**D**) systems suggests that protonation of the Bcl-xL titratable residues promotes extended loop conformations in the POPC system and compact loop conformations in the POPC:TOCL system.

To evaluate the effect of the protein protonation state and the addition of cardiolipin on the conformation of the α1-α2 loop, we computed the distribution of the radius of gyration over the same trajectory interval, from 2.25 μs to 5.0 μs, on all four simulations (Figure 4). In both the unprotonated (Figure 4C) and protonated (Figure 4D) simulation systems, the POPC:TOCL system shows broader distributions than the POPC system, indicating a more flexible loop. In the POPC simulation systems, protonation up-shifts the radius of gyration distribution, indicating an extension of the loop. Conversely, in the case of cardiolipin, protonation narrows and down-shifts the distribution, suggesting a more compact conformation.

**Figure 5:**
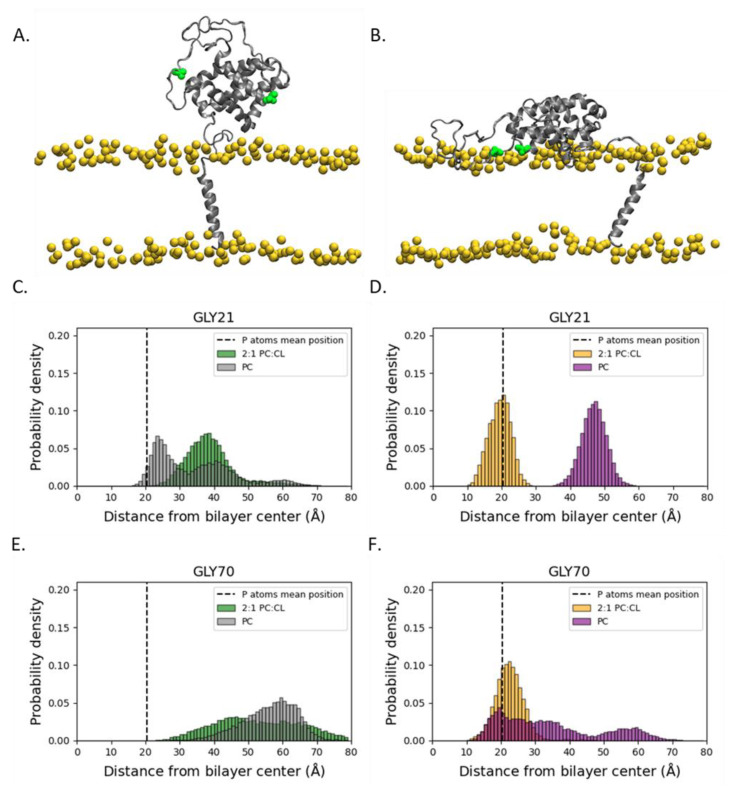
Interactions of the α1-α2 loop with the membrane surface. (**A**) Configuration snapshot corresponding to a α1-α2 loop that is not interacting with the membrane surface in the unprotonated POPC:TOCL system. (**B**) Configuration snapshot corresponding to a membrane-interacting α1–α2 loop in the protonated POPC:TOCL system. The protein is shown in secondary structure representation colored gray, while Gly-21 and Gly-70 are shown in green as filled-spheres. The lipid phosphate phosphorus atoms are shown as yellow spheres. The rest of the simulation system has been removed for clarity. (**C**–**F**). The distributions of two glycines in the α1-α2 loop (Gly-21 near α1 and Gly-70 in the middle of the loop chain) along the transmembrane direction suggest that protonation of Bcl-xL promotes the interactions of the loop with the membrane surface, while the addition of cardiolipin favors the partitioning of the loop residues in the membrane lipid headgroup region. The vertical dashed line in the histograms is located at the mean position of lipid phosphate group phosphorus atoms, an indication of the location of the membrane lipid headgroup region along the transmembrane direction.

To evaluate the membrane interactions of the α1-α2 loop, we determined the distributions of Gly-21 and Gly-70 along the transmembrane direction (Figure 5). Gly-70 was selected for this analysis due to its membrane interaction in the inserted form of Bcl-xL [15]. Its location near the middle of the 61-residue long α1-α2 loop was compared to Gly-21, which constitutes the first residue in the loop allowed an inspection of membrane interactions between different regions of the loop. In the unprotonated systems, Gly-21 has a broader distribution closer to the membrane headgroup region in the POPC simulation and a narrower distribution further from the membrane surface in POPC:TOCL (Figure 5C). Protonation of Bcl-xL results in a narrower distribution for both simulation systems. However, in the protonated POPC simulation, Gly-21 was shifted away from the membrane surface and into the bulk solvent, while in the protonated POPC:TOCL simulation, the Gly-21 distribution laid within the membrane lipid headgroup region centered at the level of the lipid phosphate groups (Figure 5D).

A similar analysis was performed on Gly-70 (Figure 5E,F) in order to evaluate the membrane interactions near the middle of the disordered loop. In the unprotonated systems, Gly-70 resides in the bulk solvent (Figure 5E), indicating that Gly-70 is not interacting with the membrane surface. In contrast, the corresponding distributions for the protonated systems suggest that Gly-70 interacts with the membrane surface upon protonation of the Bcl-xL titratable residues (Figure 5F), with a narrower distribution for protonated Bcl-xL in POPC:TOCL, centered at the level of the lipid phosphate group as in the unprotonated systems. Taken together, these results show that protonation promotes the interactions of the α1-α2 loop with the membrane surface, while the addition of cardiolipin promotes the partitioning of the loop residues in the membrane lipid headgroup region.

### 2.3. Transmembrane Helix Orientation

**Figure 6:**
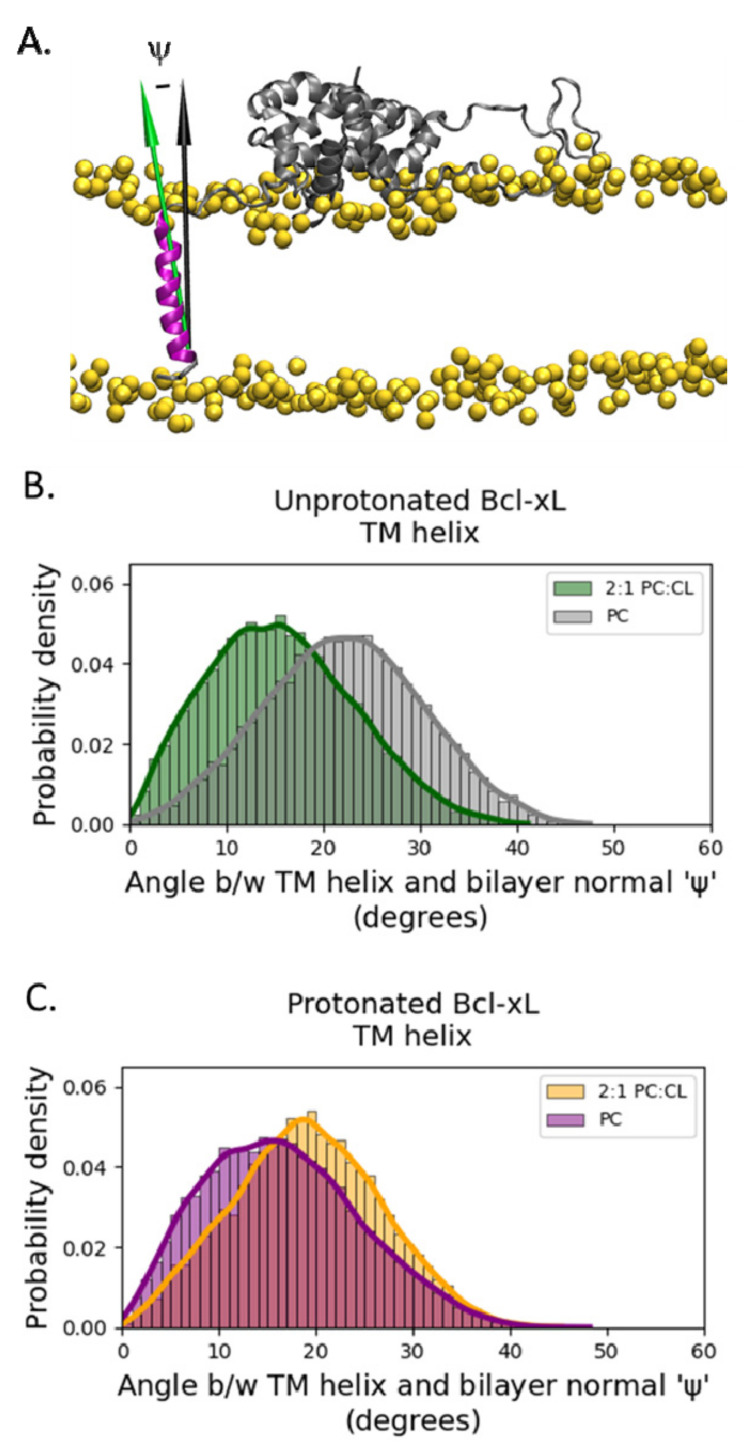
Orientation of the transmembrane (TM) helix. (**A**) The orientation of helix α8 was determined by the deviation angle, ψ, of the helix principal axis (green arrow) from the membrane normal (black arrow). The protein is shown in secondary structure representation colored gray, except for helix α8 which is shown in purple. The lipid phosphate phosphorus atoms are shown as yellow spheres. The rest of the simulation system has been removed for clarity. Distributions of TM helix angles for unprotonated (**B**) and protonated (**C**) simulations show significant overlap and provide no information correlated with soluble head orientation.

We determined the orientation of the transmembrane helix (α8) principal axis vector with respect to the membrane normal as a function of time. The corresponding distributions of deviation angles for all four systems show significant overlap (Figure 6), which suggests that neither protonation nor the addition of cardiolipin affect the conformational dynamics of α8 in a transmembrane configuration.

## 3. Discussion

Apoptosis or controlled cell death is a critically important process in maintaining and developing healthy cell populations and tissues. Dysregulation of apoptosis has been shown to have significant consequences, from hyperactive apoptosis contributing to neurodegeneration or immunodeficiency to insufficient apoptosis leading to autoimmunity and cancer [3]. Furthermore, cancer treatment efficacy is reduced by the ability of cancer cells to avoid apoptosis. A key step in triggering apoptosis is the permeabilization of the mitochondrial outer membrane, which releases apoptotic factors into the cytosol and ultimately results in cell death [22,23]. MOMP is controlled and regulated by the Bcl-2 family of proteins, which directly interact with the mitochondrial outer membrane to either promote or prevent the formation of oligomeric pores [10,11,24]. In the case of Bcl-xL, changes in membrane lipid composition [15,16,20,21], as well as the protonation state of Bcl-xL [15,16], have also been implicated in its regulation of apoptosis. While protonation changes in proteins are unlikely to be caused by decreases in cytosolic pH, several other factors modulate the pK_a_ of titratable residues. It is well known that the pK_a_ values for D and E residues have a range of 5 pH units depending on factors such as environmental polarity and water exposure [25,26]. In the case of membrane-active proteins such as Bcl-xL, these changes in pK_a_ could be induced by their water-to-membrane transitions [27,28].

The anchoring of Bcl-xL to lipid bilayers is therefore hypothesized to modulate the protonation state of its titratable residues, which could affect its membrane interactions and dynamics. Here, we examined Bcl-xL under four different conditions: protonated or unprotonated Bcl-xL embedded in a 100% POPC or 2:1 POPC:TOCL membrane.

The evaluation of the orientation of the soluble head reveals that as individual perturbations, neither the presence of cardiolipin in the membrane nor the protonation of the titratable residues result in exposure of the BH3-binding groove, suggesting that cardiolipin and protonation act in concert to promote a favorable orientation of the soluble head that exposes the BH3-binding site in the microsecond timescale (Figure 2 and Figure 3). In contrast, the soluble head orientation in the POPC unprotonated system exhibits large fluctuations over the entire conformational space allowed by the membrane-anchored configuration, which is inconsistent with the development of a competent binding conformation.

Analysis of the α1-α2 loop indicates that the addition of cardiolipin results in a more flexible loop (Figure 4). Protonation is shown to extend the loop in the presence of POPC. However, the extended loop in protonated Bcl-xL attains a narrow distribution of values in the presence of cardiolipin, suggesting a stable and extended disordered loop (Figure 4D). This is further exemplified when considering individual loop residues. While the position of Gly-21 is constrained by the orientation of the soluble head, as it is located right after helix α1, Gly-70 is near the middle of the loop and not constrained by the soluble head (Figure 5). The distributions in Figure 5E,F support the conclusion that protonation promotes membrane interactions for Gly-70, consistent with previous in vitro measurements [15].

In contrast to the rest of the protein, the orientation of the transmembrane helix with respect to the membrane normal remains largely the same across all four systems (Figure 6), with tilt angle fluctuating in the range of 10–25°. This range is somewhat lower than reported previously [13,14], which is consistent with the thicker bilayer used in our simulation.

**Figure 7:**
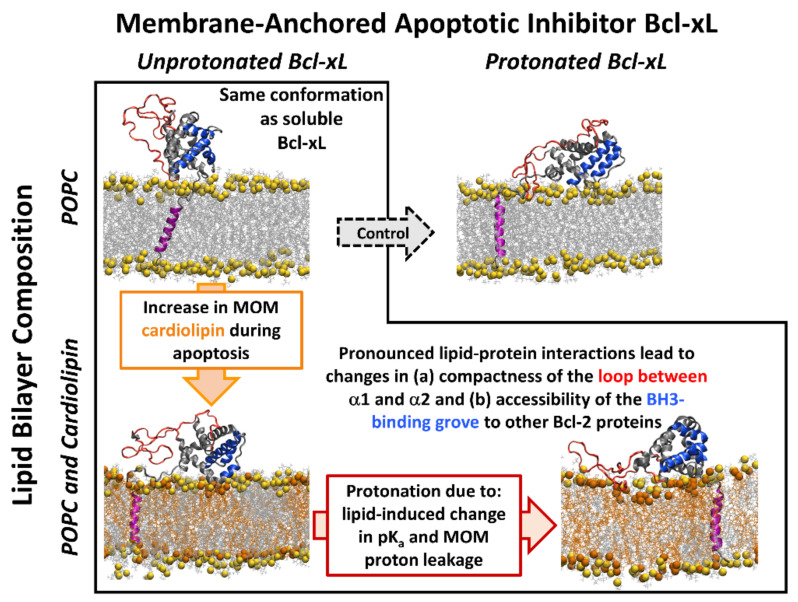
Schematic summary of the effects of cardiolipin and protonation on the conformational dynamics of membrane-anchored Bcl-xL studied by MD simulations. Four systems examined in this study are illustrated by the following representative snapshots from microsecond MD trajectories: 2.44 µs for E^−^, D^−^, and H^+^ Bcl-xL in a POPC lipid bilayer (top left); 3.66 µs for E°, D°, and H° Bcl-xL in a POPC lipid bilayer (top right); 4.30 µs for E^−^, D^−^, and H^+^ Bcl-xL in a 2:1 POPC:TOCL lipid bilayer (bottom left); and 3.71 µs for E°, D°, and H° Bcl-xL in a 2:1 POPC:TOCL lipid bilayer (bottom right). In the absence of cardiolipin or protonation (top left), the soluble head domain of the membrane-anchored Bcl-xL does not interact with the lipid membrane surface and largely retains the conformation of the soluble protein [12,13,14]. The addition of cardiolipin (bottom left, orange), which corresponds to physiologically observed changes of MOM during apoptosis [20,21], induces membrane interactions of the soluble head domain of the protein, resulting in protein conformational changes (see Figure 3, Figure 4, Figure 5, Figure 6 and Figure 7). These interactions become more prominent upon subsequent protonation of all acidic and His residues (bottom right), resulting in (i) lipid interactions of the loop between helices α1 and α2 (highlighted in red) and (ii) reorientation of the binding grove (highlighted in blue) that can engage BH3 domains of the other Bcl-2 proteins. Physiologically, the protonation corresponds to the bilayer-induced shifts in pK_a_ [15,16] or proton leakage at the early stages of MOMP [15,16]. Note that no loop-lipid binding or reorientation of the BH3-binding groove was observed in the control MD simulations of protonated Bcl-xL in the absence of cardiolipin (top right). Conformational changes and lipid interactions induced by cardiolipin and in the membrane-anchored Bcl-xL provide structural insights into the initial protein conformational changes on the pathway towards fully refolded membrane-inserted Bcl-xL [15,16].

Overall, this work provides structural insights into the dependence of the conformational dynamics of Bcl-xL on protonation states and membrane composition. Protonation and the addition of cardiolipin reorient and stabilize the soluble head in a new configuration with a high probability that the BH3-binding groove is not occluded (Figure 7). These same conditions also produce a more dynamic α1-α2 loop that readily interacts with the membrane surface, recapitulating the coupled effect of protonation and cardiolipin content (Figure 7). The concerted effects of protonation and cardiolipin on the membrane-anchored form of Bcl-xL are consistent with in vitro measurements of the Bcl-xL membrane insertion, which showed that the presence of cardiolipin produces more favorable protonation-dependent insertion free energy [15,16]. The extent to which cardiolipin modulates the protonation of Bcl-xL by altering the pK_a_ of its titratable residues is, however, a complex issue, the characterization of which will require further studies.

## 4. Materials and Methods

### 4.1. Simulation Systems

A model of the full-length Bcl-xL protein in a membrane-anchored configuration was constructed by combining two solution NMR structures: one lacking the C-terminal helix (PDB ID: 1LXL; residues 1–222) [29] and another of the isolated C-terminal helix (PDB ID: 6F46; helix α8, residues 209–231) [30]. After aligning the C-terminal helix along the Cartesian z-axis, the two structures were superimposed and joined at residue Gln-207 to generate a model for the membrane anchored full-length Bcl-xL, where the C-terminal helix is in a transmembrane orientation. For each simulation, Bcl-xL was anchored into the membrane by embedding the C-terminal helix in the lipid bilayer such that the Trp-213 residue was just below the lipid phosphate groups and the soluble folded domain was within 4 Å of the membrane surface. The membrane-anchored Bcl-xL model was incorporated into four different simulation systems, each with one of two protein protonation states to which all Bcl-xL acidic and histidine residue sidechains were protonated or unprotonated (hereafter identified as protonated or deprotonated configurations, respectively); and one of two membrane compositions: 100% 1-palmitoyl-2-oleoyl-sn-glycero-3-phosphocholine (POPC) or POPC at a 2:1 ratio with 1,1,2,2,-tetraoleoyl-cardiolipin (2POPC:1TOCL). The simulation system setup was performed with CHARMM-GUI [31,32,33,34,35,36,37]. All simulations are summarized in Table 1.

For the unprotonated 100% POPC simulation, Bcl-xL was embedded in a lipid bilayer consisting of 411 POPC, both solvated with 36,055 water molecules and neutralized with 50 mM excess NaCl (43 Na^+^, 32 Cl^−^), for a total of 166,907 atoms and an initial simulation cell size of 129.9 Å × 129.3 Å × 124.8 Å. For the protonated 100% POPC simulation, Bcl-xL was embedded in a lipid bilayer consisting of 412 POPC, both solvated with 36,094 water molecules and neutralized, and with 50 mM excess NaCl (32 Na^+^, 56 Cl^−^), for a total of 167,206 atoms and an initial simulation cell size of 130.0 Å × 128.1 Å × 125.0 Å. Similarly, for the unprotonated 2:1 POPC:TOCL simulation, Bcl-xL was embedded in a lipid bilayer consisting of 239 POPC and 120 TOCL, both solvated with 46,584 water molecules and neutralizing Na^+^ (131 Na^+^), for a total of 205,383 atoms and an initial simulation cell size of 151.1 Å × 143.3 Å × 129.4 Å. For the protonated 2:1 POPC:TOCL simulation, Bcl-xL was embedded in a lipid bilayer consisting of 239 POPC and 120 TOCL, both solvated with 46,586 water molecules and neutralizing Na^+^ (96 Na^+^), for a total of 205,389 atoms and an initial simulation cell size of 150.6 Å × 157.9 Å × 127.3 Å.

### 4.2. Molecular Dynamics Simulations

The initial equilibration of the simulation systems was performed with NAMD 2.11 [38]. Each system was subjected to 50,000 steps of conjugate gradient energy minimization, followed by a 400-ps run at constant temperature (310 K) and pressure (1 atm) with harmonic restraints one the protein-backbone atoms and the POPC carbonyl carbons using a decreasing force constant equal to 10, 5, 2, 1, 0.5, 0.2, 0.1, and 0.05 kcal∙mol^−1^∙Å^−2^. Unrestrained dynamics were then run for 10 ns on the unprotonated simulations, 7 ns on the protonated 100% POPC simulation, and 15 ns on the protonated 2:1 POPC:TOCL simulation. The CHARMM36 [39,40] force fields were used for the protein, lipids, and ions, and the TIP3P model was used for water [41]. The smooth particle-mesh Ewald summation method [42,43] was employed for the calculation of electrostatic interactions. Short-range real-space interactions were cutoff at 12 Å, employing a force-based switching function. A reversible multiple-time step algorithm [44] was used to integrate the equations of motion with a time step of 1 fs for electrostatic forces, 1 fs for short-range non-bonded forces, and 1 fs for bonded forces. A Langevin scheme was used for temperature control and a Nosé–Hoover–Langevin piston [45,46] was used for pressure control.

### 4.3. Microsecond-Timescale Simulations

After initial equilibration, all simulation systems were transferred to Anton2, a special purpose supercomputer for biomolecular simulations, and run for 5 µs each [47]. The CHARMM36 force field [39,40] was used for the protein, lipids, and ions, and the TIP3P model was used for water [41]. The r-RESPA algorithm [48] was used to integrate the equations of motions with a time step of 3 fs for long-range non-bonded forces and a 1 fs time step for both short-range non-bonded and bonded forces. The k-Gaussian split Ewald method [49] was used for the long-range electrostatic interactions. The SHAKE algorithm [50] was employed to constrain all hydrogen atom bond lengths. Simulations were performed at a constant temperature (310 K) and pressure (1 atm) using Nosé–Hoover chains [51] and the Martyna–Tobias–Klein barostat [45]. The RESPA algorithm, temperature control, and pressure control were implemented using the multigrator scheme [52].

### 4.4. Trajectory Analysis

To evaluate the orientation and accessibility of the Bcl-xL BH3-binding groove, we define a BH3-binding groove vector as the normal to the plane formed by the principal axis of helix α5 (from residue 143 to residue 153) and the vector connecting the geometric center of the α-carbons in residues 143 through 146 in helix α5 to the geometric center of the α-carbons in residues 170 through 173 of helix α6.

The α-helix principal axes were determined from the moment of inertia tensor of all the Cα atoms of all the residues participating in the helix in the initial configuration.

Analyses were performed with VMD [53] and custom Python scripts. Molecular graphics were generated with VMD.

## Figures and Tables

**Table 1:** Summary of microsecond-timescale simulations run on Anton2.

Simulated System	Protonation State	Membrane Composition
Bcl-xL with 50 mM NaCl	Protonated Bcl-xL	POPC
Bcl-xL with 50 mM NaCl	Unprotonated Bcl-xL	POPC
Bcl-xL with neutralizing NaCl	Protonated Bcl-xL	2:1 POPC:TOCL
Bcl-xL with neutralizing NaCl	Unprotonated Bcl-xL	2:1 POPC:TOCL

## Data Availability

All data and analysis scripts are available upon request.

## Supplementary Materials

The following are available online at https://www.mdpi.com/article/10.3390/ijms22179388/s1.
