# Supplementary material for: Effects of Cardiolipin on the Conformational Dynamics of Membrane-Anchored Bcl-xL
Source: Int J Mol Sci. 2021 Aug 30;22(17):9388. doi: 10.3390/ijms22179388 (PMC8431346; doi:10.3390/ijms22179388)
Supplement: Supplementary file 1 [file ijms-22-09388-s001.zip › ijms-1344116-supplementary.pdf]

Supplemental Data

Effects of Cardiolipin on Conformational Dynamics of Membrane-Anchored Bcl-xL

Vivek Tyagi [1], Victor Vasquez-Montes [2], J. Alfredo Freites [3], Alexander Kyrychenko [2,4], Douglas J. Tobias [3,*] and Alexey S. Ladokhin [2,*]

[1] Department of Materials Science and Engineering, University of California, Irvine, CA 92697, USA
[2] Department of Biochemistry and Molecular Biology, The University of Kansas Medical Center, Kansas City, KS 66160, USA
[3] Department of Chemistry, University of California, Irvine, CA 92697, USA
[4] Institute of Chemistry and School of Chemistry, V. N. Karazin Kharkiv National University, 4 Svobody Square, 61022 Kharkiv, Ukraine
* Correspondence: D. J. Tobias dtobias@uci.edu; Tel.: +1 949 824 4295
* Correspondence: A. S. Ladokhin aladokhin@kumc.edu; Tel.: +1 913 588 0489; Fax: +1 913-588-7440

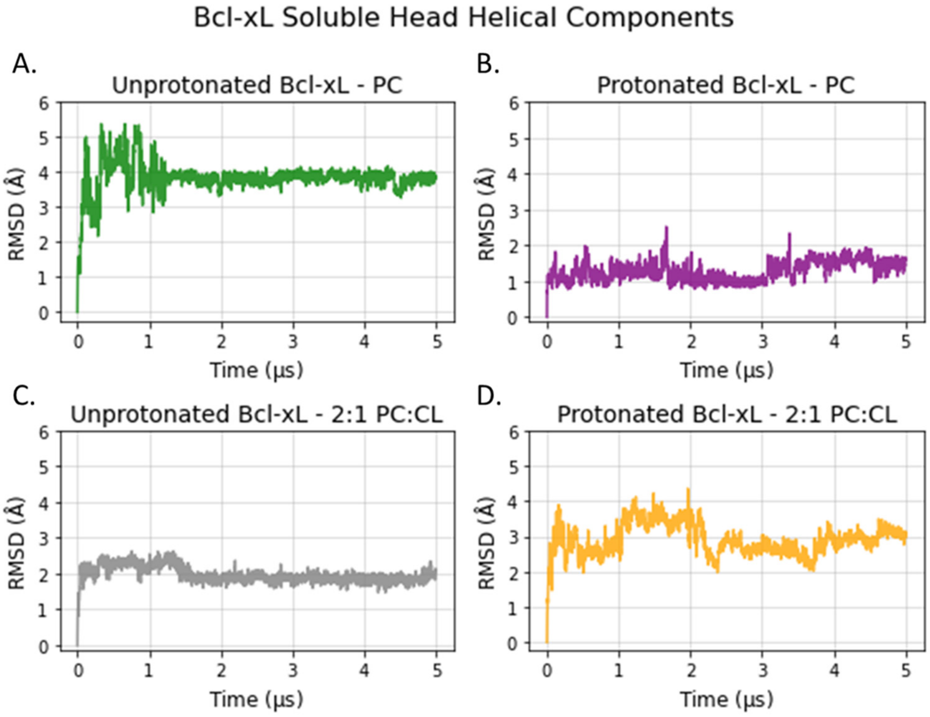

Supplemental Figure S1. RMSD of the soluble head (SH) components of Bcl-xL**.** RMSDs for all four simulated systems are shown as a function of time. RMSDs were calculated from the backbone (C, N) atoms of the helical residues in α1 through α7 without including any residues in the disordered regions linking the helices.
